# Exploration of the Esophageal Mucosal Barrier in Non-Erosive Reflux Disease

**DOI:** 10.3390/ijms18051091

**Published:** 2017-05-19

**Authors:** Nicolaas F. Rinsma, Ricard Farré, Fred J. Troost, Montserrat Elizalde, Daniel Keszthelyi, Zsuzsanna Helyes, Ad A. Masclee, José M. Conchillo

**Affiliations:** 1 Division of Gastroenterology-Hepatology, Department of Internal Medicine, School of Nutrition and Translational Research in Metabolism (NUTRIM), Maastricht University Medical Center, 6229 HX Maastricht, The Netherlands; n.rinsma@maastrichtuniversity.nl (N.F.R.); f.troost@maastrichtuniversity.nl (F.J.T.); m.elizalde@maastrichtuniversity.nl (M.E.); daniel.keszthelyi@maastrichtuniversity.nl (D.K.); a.masclee@mumc.nl (A.A.M.); 2 Translational Research Center for Gastrointestinal Disorders, Catholic University Leuven, 3000 Leuven, Belgium; ricard.farre@kuleuven.be; 3 Centro de Investigación Biomédica en Red de Enfermedades Hepáticas y Digestivas (Ciberehd), Instituto de Salud Carlos III, 28029 Madrid, Spain; 4 Department of Pharmacology and Pharmacotherapy, University of Pécs, 7623 Pécs, Hungary; zsuzsanna.helyes@aok.pte.hu; 5 János Szentágothai Research Center, University of Pécs, 7623 Pécs, Hungary; 6 MTA-PTE NAP B Chronic Pain Research Group, Faculty of Medicine, University of Pécs, 7623 Pécs, Hungary

**Keywords:** mucosal integrity, Ussing chamber, erosive esophagitis, non-erosive reflux disease, transepithelial electrical resistance, TRPV1, heartburn

## Abstract

In the absence of visible mucosal damage, it is hypothesized that the esophageal mucosal barrier is functionally impaired in patients with non-erosive reflux disease (NERD). The aim of the present study was to perform an exploratory analysis of the mucosal barrier in NERD compared to erosive esophagitis (EE) and controls. A second aim was to explore *TRPV1* gene transcription in relation to the mucosal barrier function and heartburn symptoms. In this prospective study, 10 NERD patients, 11 patients with active erosive esophagitis and 10 healthy volunteers were included. Biopsies from non-eroded mucosa were obtained for (1) ex vivo analyses (Ussing chamber) of transepithelial electrical resistance (TEER) and permeability (2) gene transcription of tight-junction proteins and transient receptor potential vanilloid subfamily member 1 (TRPV1). No differences in TEER or permeability were found between NERD and healthy volunteers, whereas TEER was lower in patients with erosive esophagitis. *TRPV1* gene transcription was not significantly different between EE, NERD and controls. Conclusions: esophageal mucosal barrier function and *TRPV1* transcription is not significantly altered in NERD patients. Future research is needed to explore other potential mechanisms that may account for the high symptom burden in these patients.

## 1. Introduction

In health, the multilayer of the squamous epithelium of the esophageal mucosa serves as a barrier and prevents contact of refluxed luminal content with nociceptive afferents and subsequent excitation, leading to heartburn. In patients with erosive esophagitis (EE), the barrier function is visibly impaired and reflux can easily reach the exposed afferents. In patients with non-erosive reflux disease (NERD), the esophageal mucosa appears normal on endoscopy and the mechanisms leading to heartburn are less clear. Assuming that heartburn in NERD is the result of increased excitation of mucosal nociceptive afferents, this is either related to increased exposure of noxious substances due to a functional or microscopic defect in the esophageal mucosal barrier function and/or related to increased sensitivity of nociceptive afferents. In addition, the central nervous system has significant modulatory effects that can either attenuate or augment nociceptive peripheral input [1,2,3].

With regard to the mucosal barrier, no conclusive answers have been provided in NERD patients thus far. Previous studies postulated that the histological finding of dilated intercellular spaces (DIS) in the basal and prickle cell layers of the mucosa of NERD patients is the manifestation of a mucosal barrier defect [4,5]. However, the pathophysiological relevance of DIS is disputed [6,7]. A more elegant and validated method to study the functionality of the mucosal barrier ex vivo is to use the Ussing chamber technique [8].

Concerning increased sensitivity of esophageal nociceptors and afferents, several studies found hypersensitivity in NERD patients not only for acid, but also for saline perfusion [9], for heat [10], and for mechanical stimuli when compared to healthy controls [11]. Although no conclusive data are present so far, acid-induced symptoms in the esophagus are believed to be mediated by the activation of the transient receptor potential vanilloid subfamily member 1 (TRPV1)—a molecular integrator for noxious stimuli, present in nociceptive afferents and possibly in epithelial cells [3,12,13]. *TRPV1* gene expression was previously found to be upregulated in the mucosa of NERD and EE patients. Upon TRPV1 activation, neuropeptide transmitters (i.e., calcitonin gene-related peptide (GGRP) and substance P (SP)) are released from sensory nerve endings, which have been related to reflux symptoms and may even increase the synthesis/release of inflammatory mediators [14,15]. Increased TRPV1 activity in the esophageal mucosa may not only lead to acid hypersensitivity, but also create a state of low-grade mucosal inflammation, that may eventually contribute to an impaired barrier function and esophageal erosions [13,16].

We hypothesized that NERD patients exhibit either an impaired mucosal barrier function and/or an upregulation of TRPV1 function that may account for heartburn symptoms. The primary aim was to further explore the mucosal barrier in NERD patients through the evaluation of ultrastructural and functional parameters, and its relationship with daily heartburn symptoms. A second aim was to explore *TRPV1* gene transcription in NERD patients in relation to the mucosal barrier function and daily heartburn symptoms.

## 2. Results

### 2.1. Study Population Characteristics

A total of 22 patients with gastroesophageal reflux disease (GERD) and 12 healthy volunteers were included in the study. One patient who was not able to stop acid suppressive therapy and two healthy volunteers with esophagitis were excluded at endoscopy in absence of any symptoms.

In the final analysis, 11 EE patients, 10 NERD patients and 10 healthy volunteers were included (Table 1). In the EE group, grade A esophagitis according to the Los Angeles classification was present in six patients; grade B in four patients and grade C in one patient. No differences in heartburn or regurgitation symptom scores were found between EE and NERD.

### 2.2. Ussing Chamber Experiment

**Figure 1:**
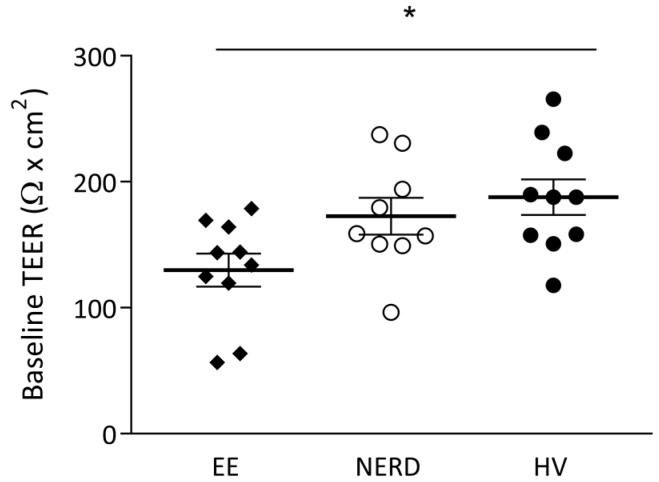
Baseline transepithelial electrical resistance (TEER) in patients with erosive esophagitis (EE), non-erosive reflux disease (NERD) and healthy volunteers (HVs). * *p* < 0.05.

In two patients (one NERD and one EE), all six biopsies were considered inadequate for reliable measurements due to insufficient aperture coverage as reflected by excessive, visible fluorescein leakage. In total, 95 biopsy samples were considered adequate for final analysis. Baseline TEER was significantly lower in EE patients when compared to healthy volunteers (139 ± 10 vs. 186 ± 11 Ω*cm^2^, *p* < 0.01) but not significantly different compared to NERD patients (175 ± 11 Ω*cm^2^, *p* = 0.08). No difference was found between the baseline TEER between NERD patients and HVs (Figure 1).

### 2.3. In Vitro Acid Exposure

**Figure 2:**
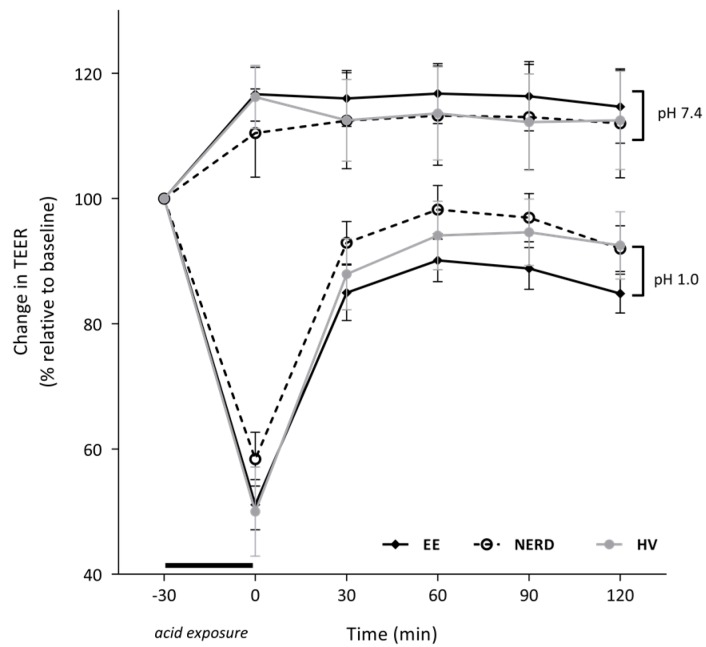
Reduction and recovery of TEER after 30 min of luminal acid exposure. Area under the curve value was larger in NERD patients when compared to EE patients (*p* < 0.05).

Replacement of the pH-neutral solution for an acidified (pH 1) KRB solution in half of the chambers for 30 min caused a gradual fall in TEER for all tissue samples (Figure 2). Other non-exposed samples served as control during the acid exposure (Figure 2). The fall in TEER was comparable between groups (EE: 48 ± 3%, NERD: 39 ± 6%, HV: 52 ± 5% relative to baseline TEER, not significant (ns). Replacement of the acidified KRB solution by a pH-neutral KRB solution after 30 min caused recovery of TEER in all tissue samples over the following 2 h. Recovery was most prominent in the first 30 min after the acid exposure, but did not reach the level of the unexposed tissue samples (Figure 2). The recovery of TEER was significantly more pronounced in NERD patients when compared to EE (AUC: 389 (347–400) vs. 310 (280–366), *p* < 0.05), however TEER recovery was not different in NERD compared to HVs.

### 2.4. Mucosal Permeability

**Figure 3:**
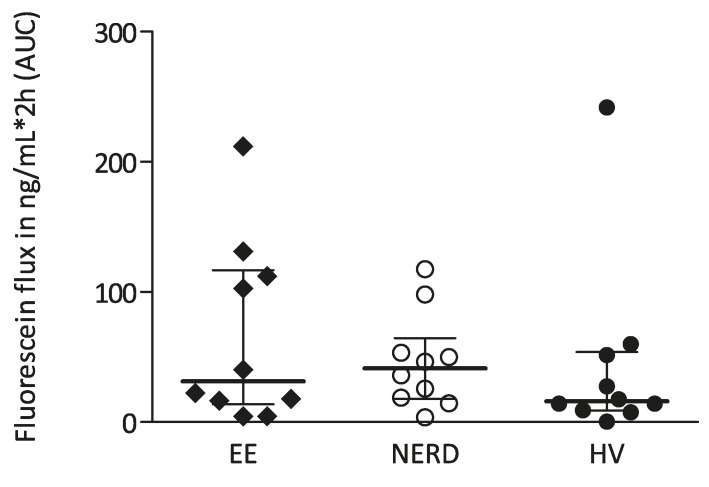
Transepithelial permeability of fluorescein (ng/mL/2h) during a 2-h period in patients with erosive esophagitis (EE), non-erosive reflux disease (NERD) and healthy volunteers (HV).

In biopsies not previously exposed to acidified KRB solution, serosal concentrations of fluorescein were very low, assuring a correct placement of the biopsy, and not significantly different between groups: AUC in ng/mL/2 h: 66.4 (22.1) in EE, 46.4 (11.5) in NERD and 44.4 (22.8) in HV (Figure 3).

### 2.5. Tight Junction Gene Transcription

Gene transcription levels of tight junction proteins (Claudin-1, Claudin-4, Occludin, E-cadherin and ZO-1) referenced to 18S rRNA gene were not significantly different between patients with EE, NERD and HVs (Table 2).

### 2.6. TRPV1 Gene Transcription

**Figure 4:**
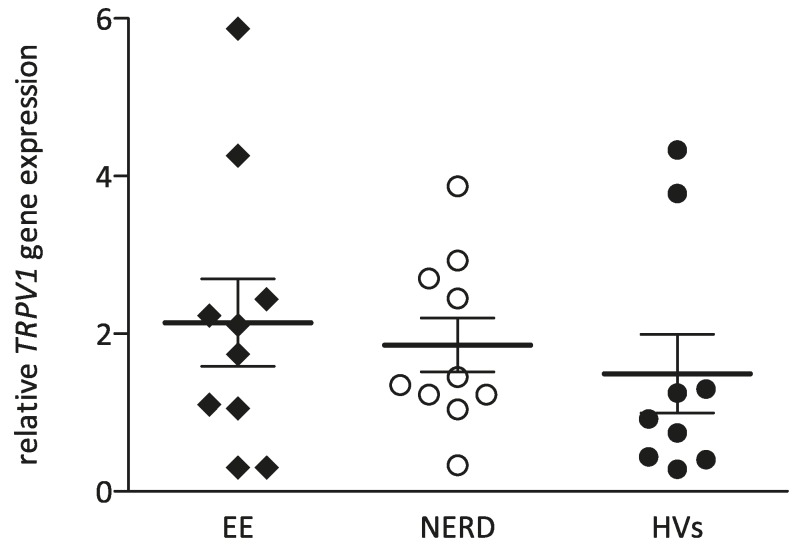
Median *TRPV1* gene transcription in patients with EE, NERD and HVs.

The level of TRPV1 gene transcription, analyzed by Q-PCR and referenced to 18S rRNA gene, was not significantly different between groups: 1.93 (0.86–2.90) in EE patients, 1.40 (1.18–2.76) in NERD patients and 0.92 (0.42–2.54) in HVs (Figure 4).

### 2.7. Associations and Correlations of Measurements

Baseline TEER was significantly correlated with permeability for fluorescein (r: −0.39, *p* = 0.04), although the correlation was moderate. TEER, nor permeability to fluorescein, was associated with *TRPV1* gene transcription. 

Reported daily heartburn severity scores, as assessed by the validated RDQ, did not show a correlation with the mucosal barrier parameters (TEER (r: −0.20, *p* = 0.31)), permeability (r: 0.01, *p* = 1.0), or *TRPV1* gene transcription (r: 0.23, *p* = 0.27).

NERD patients were divided into two groups with either normal acid exposure with a high symptom association (*n* = 4) or pathologic acid exposure (*n* = 6). Although underpowered, the groups were compared with respect to TEER (resp. 194 (102–285) vs. 155 (136–194) Ω*cm^2^, *p* = 0.38), mucosal permeability (36 (14–98) vs. 36 (15–67) ng/mL/2 h, *p* = 1.0) and intercellular space diameter (0.67 (0.55–0.70) vs. 0.84 (0.65–1.11) μm, *p* = 0.61), however no significant differences were observed.

## 3. Discussion

We performed a comprehensive and detailed investigation of the mucosal barrier in well-defined endoscopic phenotypes of GERD in order to explore the hypothesis that NERD patients exhibit an impaired mucosal barrier function and/or an increase of TRPV1 activity. While a functional defect was clearly demonstrated in the endoscopic normal mucosa of EE patients, such defects could not be found in the epithelium in NERD patients. As heartburn symptom severity was similar in both groups, these findings question the impact of an impaired mucosal barrier function in the generation of heartburn.

Our findings are in line with a recent study on the mucosal barrier function of well-defined NERD patients and the Ussing chamber [17], but also in contrast to earlier published data and current opinion on the mucosal barrier in NERD, as ultrastructural changes (dilated intercellular spaces) and low mucosal impedance values are consistently found in NERD patients. There are several reasons that may explain the discrepancy between our findings and studies supporting the role of impaired mucosal integrity in NERD.

First, most studies based the finding of an impaired mucosal barrier function, using a non-functional assessment, by evaluating the presence of dilated intercellular spaces and in vivo evaluating the mucosal impedance during ambulatory MII-pH monitoring or endoscopy [4,5,18,19,20,21]. Dilated intercellular spaces and mucosal impedance may be a surrogate for a functionally impaired mucosal barrier, however the relationship is far from clear and more conclusive studies are needed [8]. The ex vivo Ussing chamber technique determines actual permeability of ions (TEER) and larger molecules (permeability to fluorescein) across the layers of the mucosa and therefore remains the most established method for evaluation of mucosal barrier function. As the technique is laborious and handling biopsies requires experience, only two studies using the Ussing chamber on the mucosal barrier function have been performed in NERD thus far. In a study by Woodland et al., endoscopy-negative GERD patients (not defined by 24-h MII-pH monitoring and while on proton-pump inhibitors (ON-PPI) showed no difference in baseline TEER compared to healthy controls; only after an ex vivo acid challenge, TEER was more affected in the patient group [22]. Weijenborgh et al. showed that in well-defined NERD patients (with normal and abnormal acid exposure on 24-h MII-pH monitoring and off proton-pump inhibitors (OFF-PPI)), no significant differences in baseline TEER or mucosal permeability were found between NERD and healthy volunteers [17]. As was previously noted, there are serious doubts about the quality of the results of this research group due to obvious methodological pitfalls [23].

We confirmed the findings of previous studies showing that the non-eroded mucosa of patients with erosive esophagitis has an increased permeability to ions, as shown by a lower TEER [24,25]. In contrast to the ex vivo findings, in vivo mucosal impedance values in that and many other studies showed a remarkable downward gradient from healthy controls to NERD to EE [21,22,26,27,28]. This discrepancy between in vivo and ex vivo measurements in NERD patients may be explained by the possible influence of inflammatory mediators released from sensory nerve endings (SP and CGRP) and resident immune cells in the lamina propria of the mucosa [29,30]. The possible influence of these mediators on the mucosal barrier function that may be seen in vivo may no longer be present during an ex vivo evaluation of the esophageal epithelium, as the endoscopic biopsy mainly contains the epithelium. In EE patients, the presence of inflammatory mediators is more pronounced [31,32,33], and the effect of inflammatory mediators may remain present ex vivo in non-eroded areas, contributing to the damaging effects of luminal agents on the esophageal epithelium.

Secondly, the heterogeneity of NERD patients may affect study outcomes. GERD patients with normal mucosa on random endoscopy include patients with healed esophagitis, patients with abnormal acid exposure (true NERD), patients with normal acid exposure and a high symptom association probability (hypersensitive esophagus) and patients with functional heartburn [2,34]. In the present study, we made efforts to exclude patients with healed esophagitis by including an OFF-PPI period of >7 days and by reviewing previous endoscopy reports. Furthermore, patients with functional heartburn were excluded based on the symptom association analysis. The inclusion of patients with hypersensitive esophagi may have influenced group outcomes as it has been suggested that acid exposure is the major factor in impaired mucosal integrity as measured with intraluminal impedance [35,36]. In the Ussing chamber, no such correlation between acid exposure time and TEER could be established [37]. Comparison of NERD patients with and without pathologic acid exposure time in the current and previous study did not reveal significant differences in the Ussing chamber data [17], although interpretation of these comparisons due to the small subgroup sizes is rather difficult.

Study results addressing the relationship between mucosal barrier function and heartburn symptoms in NERD patients are not uniform. In the present study, mucosal barrier function did not show any correlation with reported daily life heartburn scores, which were comparable for patients with EE and NERD patients. These findings do not stand alone; in the previously mentioned study, NERD patients exhibited enhanced acid sensitivity to esophageal acid perfusion comparable with patients with erosive esophagitis, despite no clear mucosal barrier defect as assessed during endoscopy and in the Ussing chamber [17]. Furthermore, symptomatic GERD patients without erosive esophagitis during acid suppressive therapy showed no difference in baseline TEER when compared to asymptomatic, non-GERD controls [22]. On the other hand, the onset and severity of acid perception in an experimental setting during esophageal acid perfusion (Bernstein test) seems to be related to low mucosal impedance [21,38]. The question then arises whether experimentally induced heartburn symptoms reflect daily life heartburn symptoms, and what role is played by the mucosal barrier function in heartburn perception, especially in patients suffering from these symptoms on a chronic basis.

We also explored the hypothesis that heartburn in NERD patients could be due to an increased transcription of one of the principal acid sensing receptors, TRPV1, in the esophageal mucosa [3]. TRPV1 may contribute greatly to acid hypersensitivity in NERD, as TRPV1 activation by capsaicin infusion leads to heartburn [12]. Furthermore, due to the release of TRPV1-associated neuropeptides, several pro-inflammatory changes in the esophageal mucosa may occur, creating neurogenic inflammation in the esophageal mucosa that may lead to mucosal hypersensitivity. A few studies (in a limited number of patients) found higher TRPV1 expression both in NERD (as in EE patients) and TRPV1-associated neuropeptides, and have been related to symptom severity in NERD patients [14,39,40,41]. Furthermore, TPRV1 has been linked to mucosal inflammation as TRPV1-lacking mice were less prone to develop esophagitis during continuous acid exposure, in comparison with wild types [42]. We did not find a significant alteration of *TRPV*1 gene transcription in EE or NERD patients versus controls, or an association with reported symptom scores. However, a non-significant upward gradient in *TRPV1* gene transcription was observed going from healthy controls to NERD to EE. Despite the possible role of TRPV1 in esophageal nociceptive and inflammatory processes, no association between *TRPV1* gene transcription and symptoms or parameters of mucosal barrier function could be established.

A possible confounding factor is that the current assessment was performed under basal conditions: these parameters may have been different among groups during acute acid stress. Furthermore, the mucosal TRPV1 mRNA in our study is most likely to originate from non-neural cells (such as epithelial cells), as under normal conditions, there is no translation of TRPV1 in peripheral nerve terminals [43,44]. The expression of the TRPV1 protein on peripheral nerve endings, however, might be altered and more research using immunohistochemistry or Western blot analysis to determine the TRPV1 protein expression is warranted. Finally, it is well established from visceral pain studies that nociceptive input and pain perception are highly non-linear due to the pain-modulatory effects of the central nervous system, which have also been described extensively in NERD [3]. These aspects were not subject to analysis in this study.

The fact that some findings reported or assumed previously in literature could not be reproduced in the present study shows that heartburn perception in GERD is not completely elucidated and is more complex than initially expected. Nevertheless, our findings can be reconciled with esophageal pain perception. Esophageal hypersensitivity to exogenous acid in EE patients can be explained by the evident epithelial disruption, but not only in the eroded areas. Luminal contents can easily reach sensory nerves and trigger symptoms without TRPV1 upregulation. Moreover, we cannot neglect that heartburn can be triggered not only by the activation of chemoreceptors located in sensory afferent nerves near the epithelium, but also by the mechanoreceptors located in sensory nerves innervating the smooth muscle of the esophagus. It has been described by Mittal’s group that the majority of the spontaneous and acid-induced heartburn episodes in GERD patients are temporally associated with sustained esophageal contractions (SEC) of the longitudinal muscle [45,46]. Whether SEC are present and induce symptoms in NERD patients is unknown, but an altered esophageal integrity and a dysregulation of the expression of TRPV1 in epithelial cells and/or sensory nerves innervating the lamina propria is not needed in this new scenario. Moreover, we can exclude that at least some of the NERD patients have a different sensory innervation with more nerves reaching the upper layers of the epithelium that can be easily activated by acid [47].

Our study has two limitations: first, we recognize that the sample size is small, however due to the extensive and laborious Ussing chamber experiments, the inclusion of a large sample size or population is not feasible. It is possible that comparable studies in GERD patients using the Ussing chamber used similar sample sizes for the same reason [17,24]. Concerning the TRPV1 data, the small group size could explain the lack of significant outcomes. Second, as our healthy volunteers did not undergo a 24-h MII-pH monitoring, pathologic acid exposure in the absence of heartburn symptoms cannot be ruled out. However, we would like to point out that our healthy volunteers are younger than the NERD and EE patients. Although differences in TEER values and in the expression of TRPV1 receptors were not reported between younger and older populations, this may be a cofounding factor to be considered for the interpretation of the results.

In summary, this study provides a comprehensive assessment of the mucosal barrier function in well-defined endoscopic phenotypes of GERD using established methodologies. A clear mucosal barrier defect was shown in patients with erosive esophagitis, with lower TEER and a greater vulnerability to ex vivo acid exposure. In contrast, NERD patients did not show a difference in TEER or permeability when compared to controls. With similar severity of reported heartburn symptoms, this questions the relevance of mucosal barrier function in the generation of heartburn symptoms in NERD patients. Alternatively, TRPV1 activity may contribute to visceral hypersensitivity and neurogenic inflammation. Although an increasing tendency in median *TRPV1* gene transcription was observed, the differences were not significant between patients and controls.

## 4. Materials and Methods

### 4.1. Subjects

Patients with chronic (>6 months) heartburn and/or regurgitation were included from the gastroenterology outpatient clinic of the Maastricht University Medical Center. GERD patients were categorized into two endoscopic phenotypes: NERD and erosive esophagitis (EE), based on previous and study endoscopy findings. Patients with erosive esophagitis, based on previous or study-related endoscopy results, were determined as EE. Patients showing normal esophageal mucosa, based on previous and study-related upper gastrointestinal endoscopy results, but with an abnormal esophageal acid exposure (pH < 4.0 during >4% of time), or a positive association between symptoms and reflux episodes (symptom association probability (SAP) >95%) shown by 24-h pH-impedance monitoring, were categorized as NERD patients. Esophageal 24-h MII-pH monitoring was not performed per protocol; the time between study participation and 24-h MII-pH monitoring varied between 3 and 12 months. In addition to patients, we also included healthy volunteers (HVs) without a history of gastrointestinal symptoms, especially heartburn or regurgitation. Healthy volunteers were recruited at the university by advertisement and underwent a standardized screening including assessment of Rome III criteria.

Exclusion criteria for participation were: age <18 years, severe esophageal motility disorder on manometry, previous antireflux surgery, inability to stop acid suppressive medication for at least 1 week before the endoscopy, use of anticoagulants, immunosuppressive drugs and excessive alcohol consumption (>20 units per week). Written informed consent was obtained from all subjects before enrollment. This study was approved by the Medical Ethics Committee of the Maastricht University Medical Center and registered (NCT01867931, Date: 25 June 2012).

### 4.2. Study Protocol

All patients were required to stop acid suppressive therapy 7 days before the endoscopy. During upper gastrointestinal endoscopy, the esophagogastric junction was first closely inspected for the presence of erosions. Subsequently, nine biopsies were obtained approximately 5 cm above the gastro-esophageal junction using standard biopsy forceps (Radial Jaw 4 forceps, Boston Scientific, Natick, MA, USA) by an experienced gastroenterologist (JMC) using the same biopsy technique. In patients with erosive esophagitis, biopsies were taken in non-eroded areas.

### 4.3. GERD Symptom Evaluation

All patients were asked about their GERD symptoms using the validated Reflux Disease Questionnaire (RDQ) during the week without acid suppressive medication [48]. The RDQ assesses the frequency and severity of 11 GERD-related symptoms on a 5-point Likert scale.

### 4.4. Ussing Chamber Experiment

Six biopsies were directly immersed in a pre-oxygenated, iced Krebs–Henseleit buffer (KRB) solution and transferred within 5 min to a mini-Ussing chamber system (Harvard Apparatus Inc., Holliston, MA, USA). By mounting the biopsies in the Ussing chamber, a serosal and luminal compartment was created, separated by esophageal epithelial tissue (Supplementary Material S1 for detailed protocol description). A stereomicroscope was used to ensure correct orientation of the biopsy and biopsy integrity. After mounting, tissue samples were equilibrated for 40 min to achieve steady-state transepithelial electrical resistance (TEER). TEER is defined as measuring the resistance that exerts the epithelium against the passage of ions. In other words, TEER is a reliable measurement to determine the permeability of the epithelium to ions. Baseline TEER was assessed at this time point for all biopsies. Subsequently, half of the biopsies were exposed on the luminal side to an acidified KRB glucose solution (pH 1) for 30 min. Changes in TEER relative to baseline were calculated at the end of the 30-min exposure period. Consecutively, the acidic solution was replaced by a pH-neutral KRB glucose solution and the recovery of TEER was monitored during a consecutive 2-h period. The other, non-exposed biopsies served as controls and were used to assess the permeability to fluorescein (376 Da, Sigma Aldrich, St. Louis, MO, USA) at a concentration of 1 mg/mL over 120 min. It is of utmost importance to ensure correct biopsy placement for obtaining reliable outcomes from Ussing chamber experiments, as was commented in response to recent published data [23]. Biopsies with an abnormal baseline TEER of less than 50 Ω*cm^2^ or showing visual or excessive leakage of fluorescein were excluded from further analysis, as the aperture of the Ussing chambers is likely to be not fully covered or tissue viability cannot be guaranteed [22,23].

### 4.5. Tight Junction Protein and TRPV1 Gene Transcription

Transcript levels of tight junction proteins (Claudin-1, Claudin-4, Occludin, E-cadherin and ZO-1) were determined by a quantitative polymerase chain reaction (Q-PCR) using primers as described in the Supplementary Material. Q-PCR was also used to assess TRPV1 mRNA expression in the esophageal mucosa as previously described [49] (see also Supplementary Material S2). The final results are expressed as the normalized ratio between the investigated gene and reference gene 18S rRNA.

### 4.6. Data and Statistical Analysis

A sample size analysis was performed using TEER as the main parameter for mucosal barrier function. Data for executing sample size calculation were retrieved from Jovov et al. [24], in which human esophageal biopsies (from GERD patients and controls) were subjected to an Ussing chamber experiment, and a pilot test conducted at our laboratory. Calculation was executed using the following parameters for analysis of variance (ANOVA): Mean 1 (EE): 140 Ω*cm^2^; Mean 2 (NERD): 140 Ω*cm^2^; Mean 3 (healthy controls): 240 Ω*cm^2^; standard deviation: 60 Ω*cm^2^; α: 0.05; β (power): 0.8. This calculation resulted in a sample size of 9 per group. Continuous parametric variables are expressed as mean ± standard error of the mean (SEM). Non-parametric variables are expressed as median, with interquartile ranges (IQR). Analyses of Ussing chamber experiments, and *TRPV1* gene transcription were performed with one-way analysis of variance (Tukey post hoc test). A *p*-value below 0.05 was considered statistically significant. All statistical analyses were performed using commercially available computer software (IBM SPSS Statistics for Windows, Version 22, IBM Corp., Armonk, NY, USA).

## 5. Conclusions

We conclude by using several techniques whereby NERD patients do not exhibit a clear impaired mucosal barrier function and mucosal TRPV1 transcription is not significantly altered. Both factors did not interrelate nor correlate with daily heartburn symptoms. Therefore, future research should be widened on both peripheral and central pain processing mechanisms in NERD.

## Figures and Tables

**Table 1:** Group characteristics.

	EE *n* = 11	NERD *n* = 10	HV *n* = 10
Age (range) years	63 (45–77)	48 (28–70)	35 (21–65)
Male:female ratio	6:5	7:3	5:5
Esophagitis on study endoscopy	11/11	0/10	0/10
PPI-use	11/11	10/10	0/10
Heartburn score (SD)	2.8 (1.6)	2.7 (2.3)	0 (SD 0)
Regurgitation score (SD)	2.6 (1.5)	2.3 (2.0)	0 (SD 0)
Pathologic acid exposure time (>4%) on 24-h MII-pH monitoring	n/a	6/10	n/a

EE = erosive esophagitis; NERD = non-erosive reflux disease; HV = healthy volunteers; PPI = proton pump inhibitor; SD = standard deviation; n/a = not applicable.

**Table 2:** Normalized expression ratio of tight junction proteins.

	EE	NERD	HVs	*p*-Value
Claudin-1	1.28 (0.32)	1.80 (0.29)	1.25 (0.28)	0.35
Claudin-4	0.93 (0.15)	1.02 (0.34)	0.93 (0.21)	0.88
Occludin	1.70 (0.35)	1.92 (0.35)	1.47 (0.17)	0.53
E-cadherin	1.25 (0.38)	1.18 (0.37)	1.23 (0.23)	0.99
ZO-1	2.42 (0.38)	2.02 (0.37)	1.99 (0.23)	0.61

## Supplementary Materials

Supplementary materials can be found at www.mdpi.com/1422-0067/18/5/1091/s1.
