# Supplementary material for: Exploration of the Esophageal Mucosal Barrier in Non-Erosive Reflux Disease
Source: Int J Mol Sci. 2017 May 19;18(5):1091. doi: 10.3390/ijms18051091 (PMC5455000; doi:10.3390/ijms18051091)
Supplement: Supplementary file 1 [file ijms-18-01091-s001.pdf]

International Journal of
Molecular Sciences

SUPPLEMENTARY MATERIAL

S1 Ussing Chamber protocol

Esophageal biopsies were mounted in Ussing Chambers with a 9-mm opening, that was reduced to fit biopsy specimens to an area of 1.76 mm^2 using a technique previously described [47]. Both compartments were filled with 1.5 ml 10 mmol/l glucose KRB solution. The chambers were kept at 37 °C and continuously oxygenated and circulated by gas flow (95% O_2/5% CO_2) during the whole experiment. A four-electrode system was used, as described previously [48]. Ag/Cl electrodes with 3M NaCl / 2% agar bridges were used for measurement of transepithelial potential difference (PD) and current (I) monitoring. Every minute, direct pulses of 1.5, -1.5, 3.-3 and 0 µA, with duration of 235 ms were calculated. Every minute the TEER was obtained according to Ohm's law from the slope of the I-U line.

S2 TRPV1 and tight junction gene transcription

Gene transcription of 18S was used as reference. Total RNA was isolated from snap-frozen biopsies using Trizol (Invitrogen, Carlsbad, CA, USA), followed by a cleaning step with the RNeasy Mini kit (Qiagen, Austin,TX, USA). Quantification of the purified RNA was carried out using the NanoDrop spectrophotometer (Nano- Drop Technologies, Wilmington, DE, USA). C-DNA was synthesized using the iScript cDNA Syntheses Kit (Bio-Rad, Hercules, CA, USA). PCR amplifications were run on the MyIQ real-time PCR detection system (Bio-Rad, Hercules, CA, USA). PCRs were performed with 5 µL of

cDNA template diluted to a concentration of 4 ng/mL, 12.5 µL iQ Sybr Green supermix (Bio-Rad), 1 µL forward and reversed primers (10 µmol/L) and 5.5 µL of sterile water. Cycling conditions were as follows: 3 min at 95 °C for denaturation of cDNA followed by 40 amplification cycles of 10 s at 95 °C and annealing for 45 s at 60 °C.

The following primers were used:

Name	Forward / Reverse Primer Name	Sequence
**18SRNA**	18SrRNA1F<br>18SrRNA1R	5′-GTAACCCGTTGAACCCCATT-3′<br>5′-CCATCCAATCGGTAGTAGCG-3′
**ZO-1**	ZO-1 F<br>ZO-1 R	5′-AGGGGCAGTGGTGGTTTTCTGTTCTTTC-3′<br>5′-GCAGAGGTCAAAGTTCAAGGCTCAAGAGG-3′
**OCLN**	OCLNf1<br>OCLNr1	5′-TCAGGGAATATCCACCTATCACTTCAG-3′<br>5′-CATCAGCAGCAGCCATGTACTCTTCAC-3′
**CLDN-4**	CLDN4f1<br>CLDN4r1	5′-ACAGACAAGCCTTACTCC-3′<br>5′-GGAAGAACAAAGCAGAG-3′
**CLDN-1**	CLDN1_f<br>CLDN1_r	5'-GGGCTGCAGCTGTTGGGCTT -3'<br>5' GGGTTGCTTGCAATGTGCTGCT-3'
**E-cadherin**	E-cadh_f<br>E-cadh_r	5'-CACCTGGAGAGAGGCCGCGT-3'<br>5'-AACGGAGGCCTGATGGGGCG-3'
**TRPV-1**	TRPVf1<br>TRPVr1	TCTCACCTACATCCTCCTGCTCAA<br>TTGCTCTCCTGTGCGATCTTGT
